# A mobile pleural calcification

**DOI:** 10.36416/1806-3756/e20260449

**Published:** 2026-11-09

**Authors:** Edson Marchiori, Bruno Hochhegger, Gláucia Zanetti

**Affiliations:** 1. Universidade Federal do Rio de Janeiro, Rio de Janeiro (RJ) Brasil.; 2. University of Florida, Gainesville (FL) USA.

A 77-year-old asymptomatic woman was referred for evaluation of a pulmonary nodule seen on a chest X-ray during a routine check-up. Physical examination and laboratory test results were unremarkable. Chest CT demonstrated the presence of a calcified nodule in the left pleural cavity (Figure 1A). Previous chest CT scans performed 5 years earlier showed the nodule in a different position, showing that the nodule was mobile (Figure 1B). This finding was considered to be diagnostic of thoracolithiasis.

**Figure 1:**
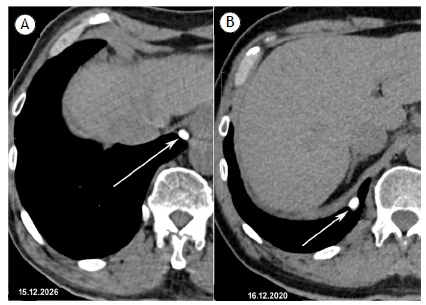
Axial chest CT image (A) showing a calcified nodule at the base of the right hemithorax, adjacent to the mediastinum (arrow). A previous chest CT performed 5 years earlier (B) showed the nodule in a different position (arrow).

Thoracolithiasis is a rare benign condition characterized by the presence of one or more mobile free bodies, with or without calcification, in the pleural space. Thoracolithiasis is rarely symptomatic, and most cases are found incidentally on radiographs or CT scans, or during surgery or autopsy. The diagnosis of thoracolithiasis can be based on imaging findings, especially the demonstration of mobility of a calcified pleural nodule. ^1^ ^-^ ^4^

Larger pleural stones can be seen on plain radiographs, but CT plays the most central role in diagnosis. The stones are usually ovoid or round and smoothly marginated, with different locations on serial scans due to mobility. Mobility, as demonstrated by sequential imaging studies, is the most characteristic finding of thoracolithiasis. Although most nodules are observed to be mobile during the course of follow-up, some are immobile and difficult to diagnose. Most stones are calcified, with or without low central density due to the presence of fat. Calcification can be diffuse and homogeneous, peripheral with an eggshell-like appearance, or spotty and central. Less-calcified or immobile thoracolithiasis is clinically important because it is often misdiagnosed and unnecessarily surgically resected. Moreover, some thoracoliths have been reported to enlarge on follow-up and may be difficult to distinguish from neoplastic disease. The differential diagnosis of a mobile pleural nodule includes fibrin bodies, foreign body granulomas, and gallstone spillage into the pleural space following cholecystectomy. Histopathologically, fibrous tissue, with or without hyalinization, surrounding a central core is found in most cases. The composition of the central core is variable, but most cases exhibit fat with or without calcification or necrosis.^1^ ^-^ ^4^

Thoracolithiasis does not require any specific treatment, especially surgical resection. For patients with mobile pleural calcified nodules, clinicians and radiologists should recommend clinical observation, as nodule removal might not be necessary for diagnosis.
